# The Effects of Temporary Interruption of Hepatic Blood Circulation

**DOI:** 10.7759/cureus.81941

**Published:** 2025-04-09

**Authors:** Tamar Turmanidze, Ketevan Jandieri, Leila Jandieri, Levan Benashvili, Revaz Otarashvili, Evgeni Asatiani, Grigol Sulaberidze, Gigi Gorgadze, Liana Kikalishvili

**Affiliations:** 1 Department of Clinical Anatomy and Operative Surgery, Tbilisi State Medical University, Tbilisi, GEO; 2 Department of Dynamic Anatomy, Tbilisi State Medical University, Tbilisi, GEO; 3 Faculty of Medicine, Tbilisi State Medical University, Tbilisi, GEO

**Keywords:** blood circulation, cholestasis, hepatocytes, liver, necrosis

## Abstract

Large-scale liver surgeries often require the temporary interruption of hepatic blood circulation, a process that can lead to potentially irreversible damage to the liver and other internal organs. This study aims to investigate the macro-morphological, histological, and ultrastructural changes in the liver and other organs following temporary hepatic blood flow interruption.

Experiments were conducted on 26 rats, randomly assigned to two groups: Group I (control: five rats) and Group II (rats that underwent bile duct ligation: 21 rats). On day 6, the hepatoduodenal ligament was ligated. Decapitation was performed at specific time points following ligature removal: 15 minutes (n = 7), 24 hours (n = 7), and 48 hours (n = 7). Tissue samples were collected for general morphological, histochemical, and electron microscopic analysis.

The investigation revealed that damage to the liver and other organs after temporary hepatic blood flow interruption and subsequent restoration was more prolonged and severe in the presence of cholestasis than in its absence.

Fifteen minutes after the ligature removal, new bile duct formation or “neoductulogenesis” was observed, creating an additional reservoir for congested bile. At 24 and 48 hours, a marked decrease in neoductule numbers was noted. This was attributed to the flow of congested bile into the bloodstream through new pathways, drainage of intrahepatic bile ducts, and reduced intrahepatic bile pressure. These changes precipitated hepatocyte metaplasia into ductal biliary epithelial cells and promoted further neoductulogenesis.

## Introduction

Large-scale liver surgeries and the prophylaxis of anticipated hemorrhage often require the temporary interruption of hepatic blood circulation. This procedure carries certain risks, most notably the development of potentially irreversible damage to the liver and other internal organs [1-3].

In the presence of cholestasis, temporary disconnection of hepatic blood flow and its subsequent restoration at different lengths of time can lead to significant injury not only in the liver but also in other organs such as the intestines, pancreas, and spleen. These procedures are typically performed in the context of underlying liver pathology, which itself contributes to the extent of tissue damage [4,5].

Thus, cholestasis most commonly arises in pathological conditions associated with liver diseases that necessitate large-scale surgery and temporary interruption of hepatic blood flow.

This study aims to evaluate the macro-morphological, histological, and ultrastructural changes in the liver and other organs (such as the intestines, spleen, and pancreas) following temporary interruption and subsequent restoration of hepatic blood circulation under cholestatic conditions. It specifically investigates the influence of cholestasis on post-reperfusion changes and explores whether systemic circulatory disturbances, rather than ischemic liver injury alone, are the primary drivers of organ damage and homeostatic disruption. Observations were conducted at 15 minutes, 24 hours, and 48 hours after reperfusion, time points identified as critical based on preliminary findings indicating significant progressive damage within these intervals.

## Materials and methods

Experiments were conducted on 26 adult white Wistar rats weighing 200-250 g. All rats were housed under standard laboratory conditions with ad libitum access to food and water. The study protocol was approved by the institutional ethical committee.

The sample size (n = 26) was determined based on power calculations and previous experimental studies evaluating hepatic ischemia-reperfusion injury. This number was sufficient to detect significant differences in morphological and ultrastructural changes across the selected time points.

The animals were randomly assigned into two groups. Group I underwent surgery with hepatoduodenal ligament ligation only, serving as the control group (n = 5). Group II underwent common bile duct ligation to induce cholestasis, serving as the cholestasis group (n = 21).

Surgical procedure

Cholestasis was induced under general anesthesia with isoflurane (5% for induction and 2%-3% for maintenance, mixed with oxygen). Body temperature was maintained at 37°C using a heating pad. The abdominal hair was shaved, and the skin was disinfected with betadine followed by 70% ethanol. A midline abdominal incision (~4 cm) was made along the linea alba using surgical scissors. The abdominal cavity was expanded with a retractor, and the liver was exposed by gently retracting the intestines caudally. The common bile duct was then identified and isolated. Local anesthesia (0.25% novocaine) was applied to the hepatoduodenal ligament.

**Figure 1:**
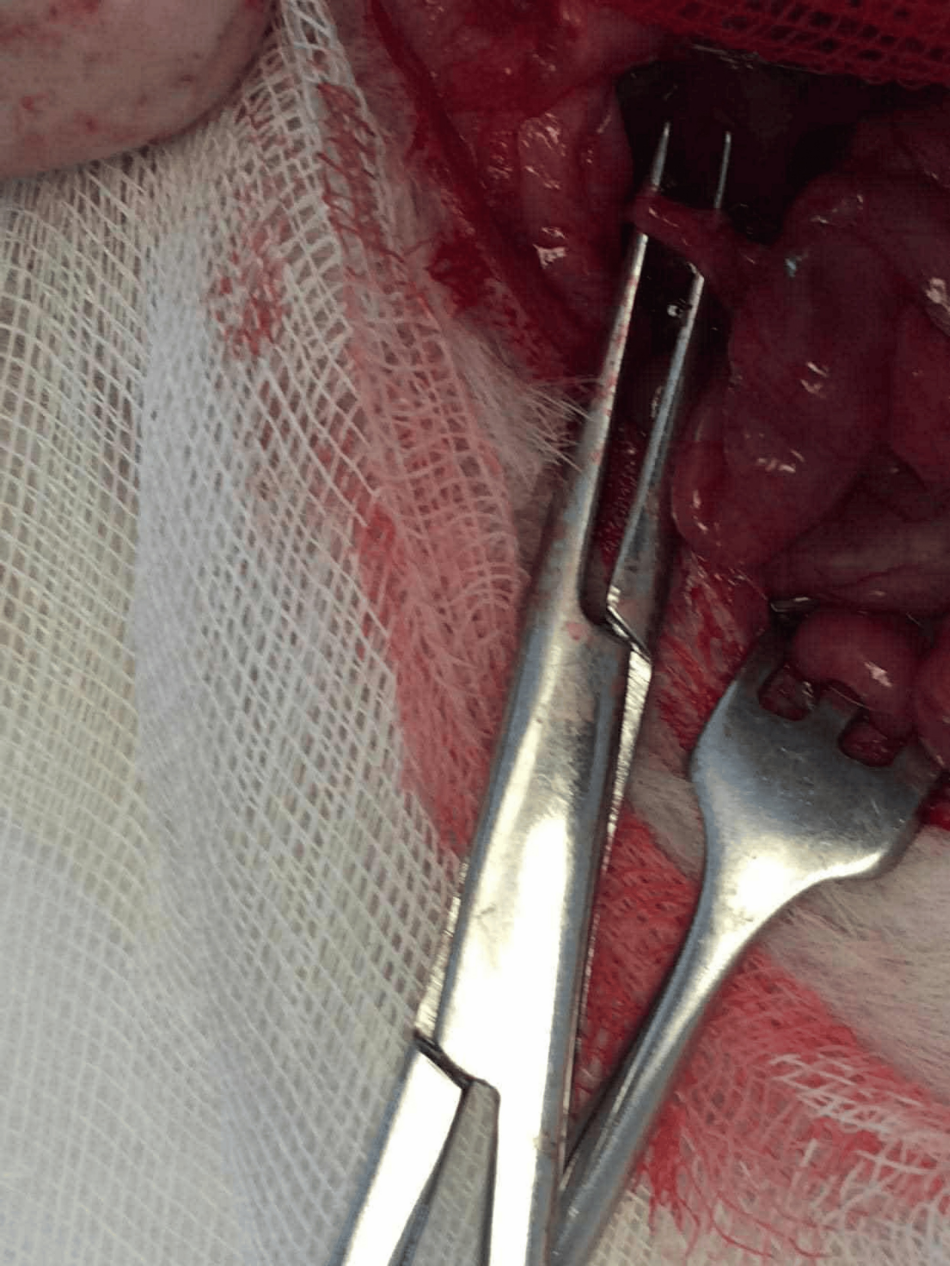
The bile duct separated from the portal vein and hepatic artery using micro-forceps.

**Figure 2:**
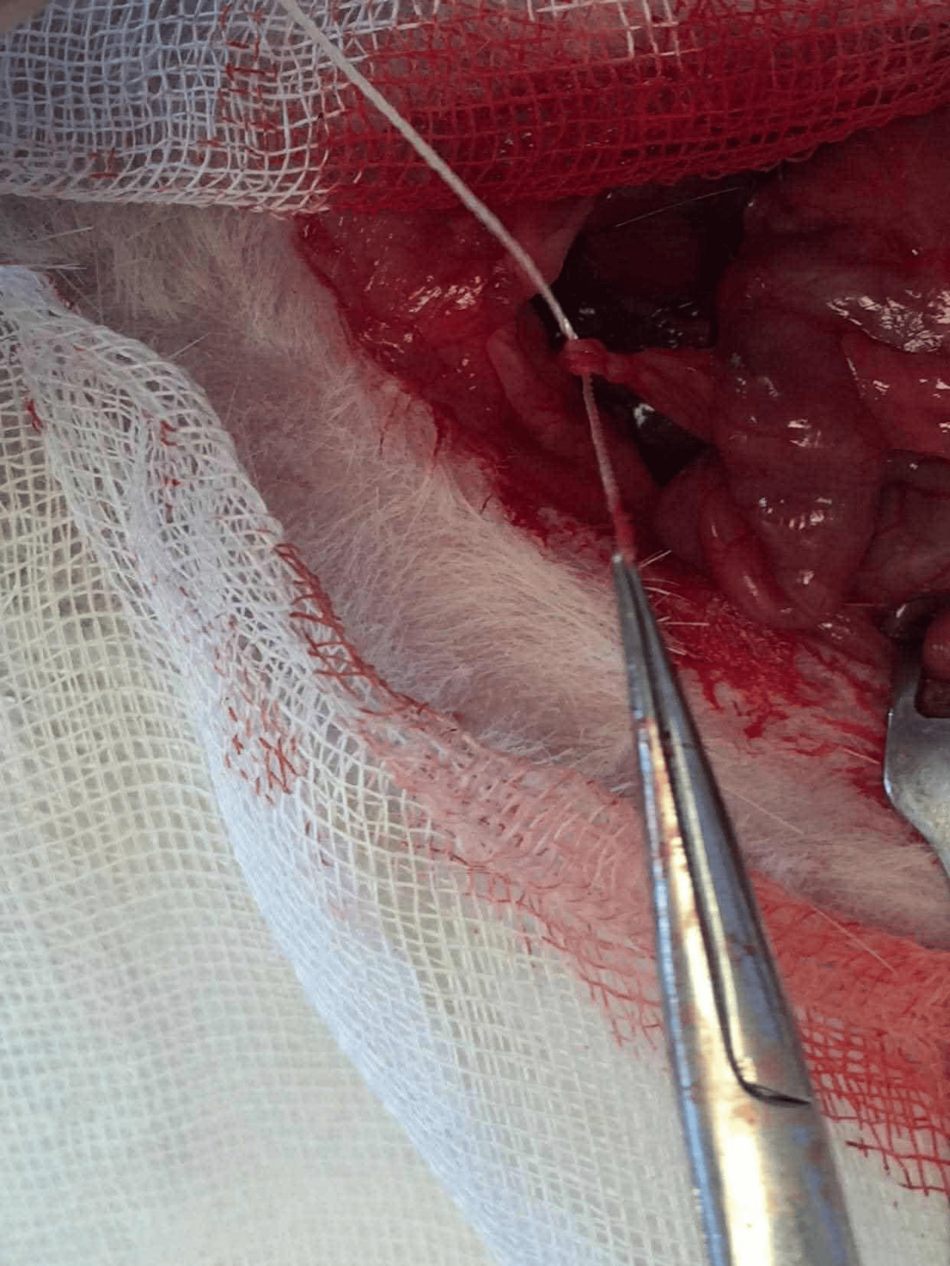
Ligature of 6.0 silk thread being placed around the bile duct.

The bile duct was carefully separated from the portal vein and hepatic artery using micro-forceps (Figure 1). Two ligatures of 6-0 silk thread were placed around the bile duct, and the segment between the ligatures was dissected (Figure 2). The abdominal cavity was then closed in layers using knotted sutures. Successful induction of cholestasis was confirmed by dilation of the bile duct.

Study timeline and time points

On day 6 post-surgery, the hepatoduodenal ligament was temporarily ligated to induce hepatic ischemia. After 15 minutes, the ligature was removed to allow reperfusion. Animals were sacrificed at the defined time points to assess damage to liver and other organs: 15 minutes (n = 7), 24 hours (n = 7), and 48 hours (n = 7) after reperfusion. These specific time points were selected based on preliminary observations indicating the onset and progression of significant tissue damage. The study aimed to evaluate both the acute and prolonged effects of reperfusion injury under cholestasis.

Tissue collection and processing

After euthanasia by decapitation, tissues from the liver, intestines, spleen, and pancreas were harvested for further analysis. The tissues were processed for the following examinations.

Macro-morphological Assessment

A gross examination of organ changes was conducted.

Histology

Tissue samples were fixed in 12% neutral formalin, embedded in paraffin, and sectioned at a thickness of 4-5 µm using a rotary microtome. Sections were stained with hematoxylin and eosin (H&E) for general morphological evaluation.

Histochemistry

Glycogen content was assessed using the Shabadash method.

Electron Microscopy (Ultrastructural Examination)

After standard processing, tissue blocks were embedded in Epon and polymerized at 60°C for 48 hours. Ultrathin sections were prepared using an LKB or LKB III ultramicrotome (Leica Microsystems, Wetzlar, Germany) and mounted on palladium grids. Contrast staining was performed with 5% uranyl acetate in absolute methanol (for 15 minutes at 45°C), followed by Reynold's lead citrate staining. Samples were examined using Tesla-100 (TESCAN, Brno, Czechia), Hitachi-100 (Hitachi High-Tech, Tokyo, Japan), and Tesla-135-500 (TESCAN, Brno, Czechia) electron microscopes.

Immunohistochemistry (Liver Only, 15 Minutes After Reperfusion)

Monoclonal antibodies were used to detect specific epithelial and proliferating cells. AE1/AE3, a pan-cytokeratin cocktail (ab961, Abcam, Cambridge, UK), was used to identify epithelial cells. OV-6 (MAB2020, R&D Systems, Minneapolis, MN, USA) was employed to detect hepatic progenitor cells. Ki-67 (ab16667, Abcam, Cambridge, UK) served as a marker for cell proliferation.

Control Group

Group I served as the control group, where no bile duct ligation was performed, allowing for comparison with the cholestasis-induced changes observed in Group II.

Histological and ultrastructural assessment criteria

Histological evaluation was based on the degree of tissue damage, including inflammation, necrosis, and cellular structural alterations. Ultrastructural assessments were focused on mitochondrial damage, endoplasmic reticulum swelling, and other features of ischemia-reperfusion injury. Immunohistochemical markers were used to assess cell proliferation and epithelial integrity.

## Results

Changes observed 15 minutes after restoration of blood circulation

Upon opening the abdominal cavity, the common bile duct was macroscopically enlarged and filled with bile. The liver was significantly enlarged, but no noticeable changes were observed in other organs.

After emptying the common bile duct, a ligature was applied to the hepatoduodenal ligament for 10 minutes. Congestion was observed in the portal system. The gastrointestinal organs turned bluish-cyanotic, the spleen was enlarged more than five times, and the liver appeared grayish-chestnut in color.

Fifteen minutes after the ligature was removed, the liver turned purple, the spleen began to return to its original size, and small hematomas were observed in the mesentery of the small intestine.

**Figure 3:**
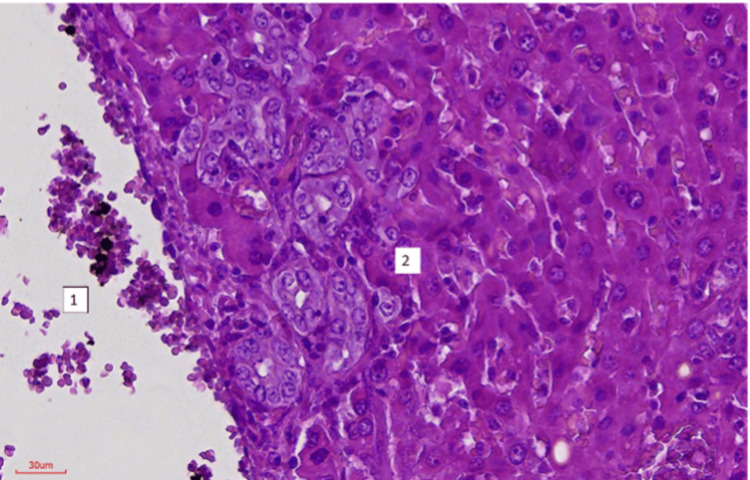
(1) Portal vein and (2) ductular reaction (H&E stain: 10x40).

**Figure 4:**
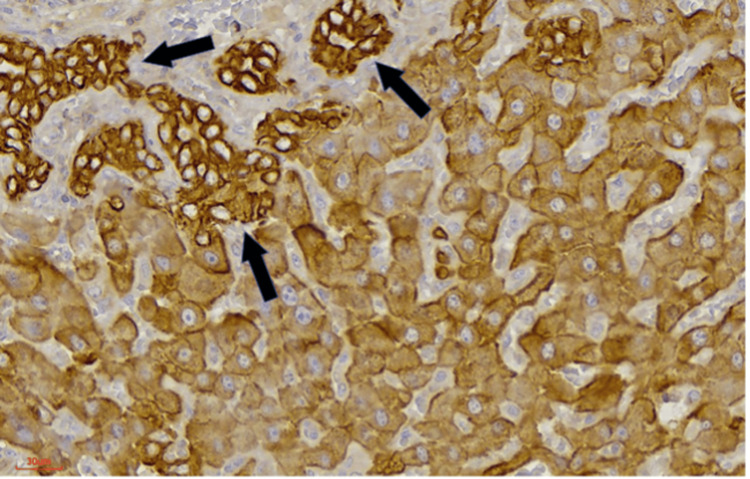
Neoductules (arrows; AE1/AE3 stain: 10x40).

**Figure 5:**
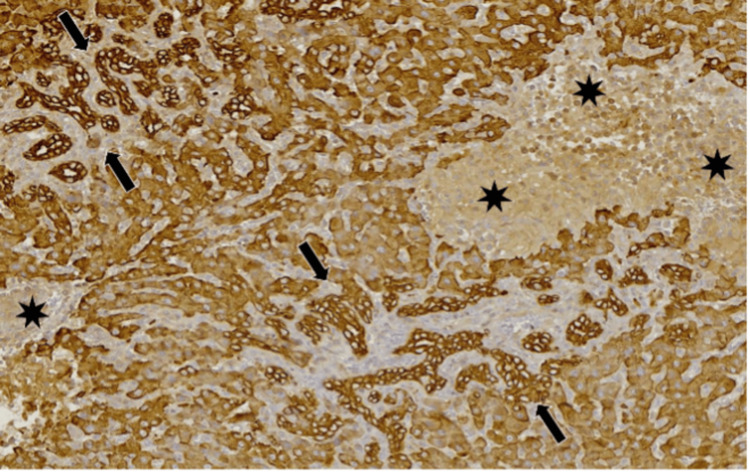
Neoductules (arrows) and bile duct necrosis (stars) (AE1/AE3 stain: 10x20).

In H&E-stained preparations, the micromorphological structure of the liver was preserved. The spaces of Disse were dilated, and the hepatocytes exhibited large- and small-droplet lipid dystrophy. Small and relatively large foci of coagulative necrosis were visible at the center and periphery of the lobules, containing eosinophilic detritus infiltrated by leukocytes. Additionally, there was a noticeable proliferation of bile capillaries, or the so-called neoductules. Glycogen was nearly absent, detectable only in a few hepatocytes, mainly at the periphery of the lobules(Figures 3-5).

**Figure 6:**
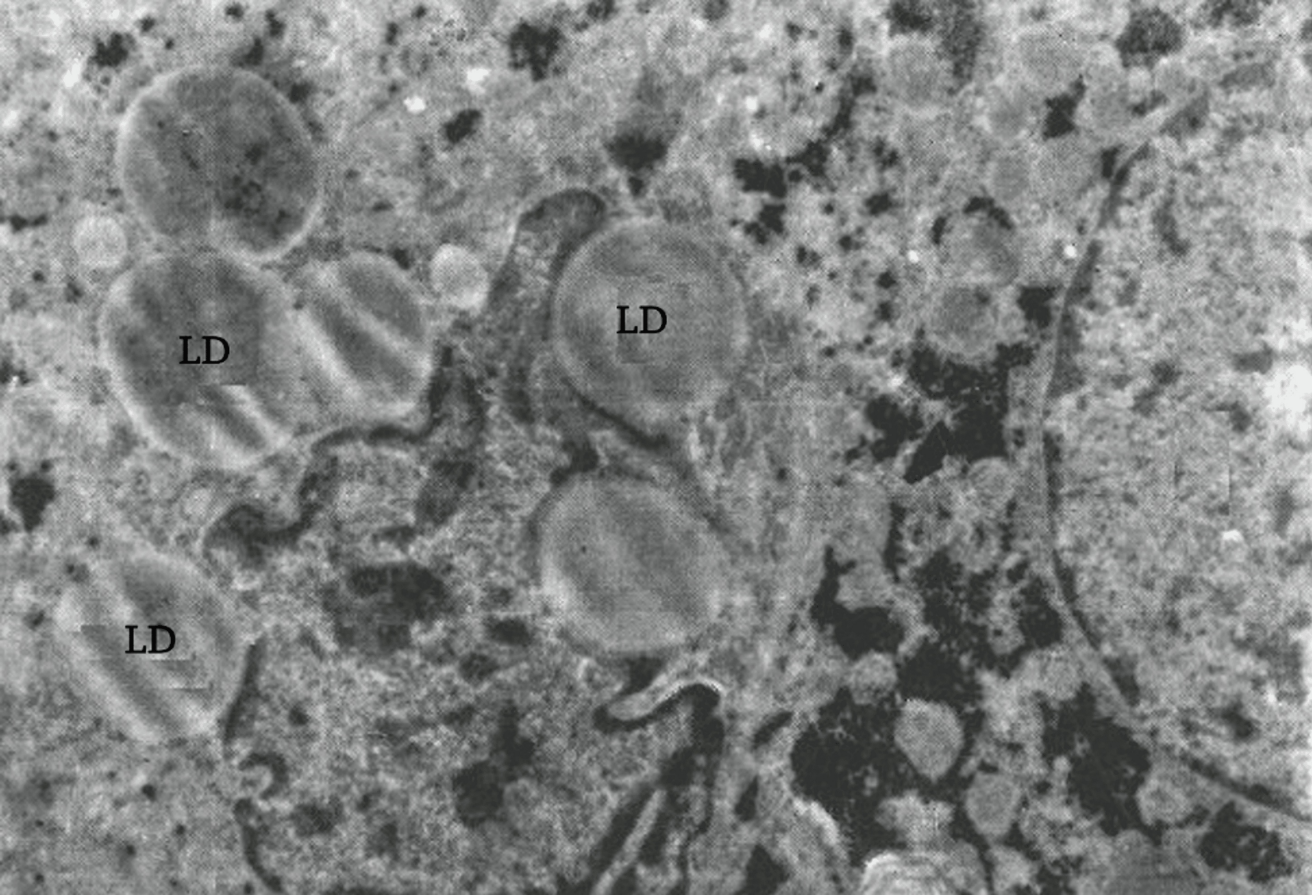
Cytoplasmic lipid droplets (LDs) visible in the hepatocytes (electron microscope: x15,000).

Electron microscopy revealed swollen hepatocytes, with dissociation observed in some areas. Lipid droplets were visible in the hepatocytes (Figure 6).

Separated pyknotic nuclei and apoptotic, necrotic hepatocytes are visible. The spaces of Disse are dilated, with single erythrocytes present within them. Erythrocytes are also detected in the central veins, where some erythrocyte sludges (clusters) are observed.

The intestinal villi are swollen and depopulated of epithelium. As a result, the crests of some villi are stripped. Blood plaques and desquamated epithelium are observed in the intestinal lumen.

**Figure 7:**
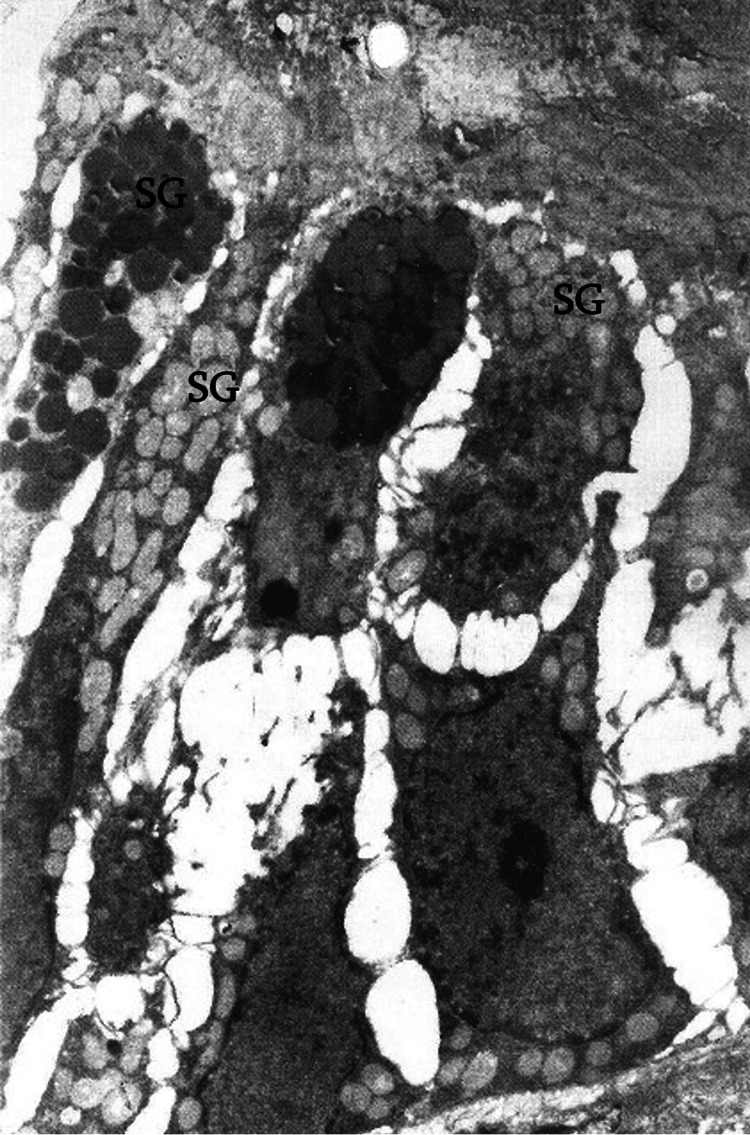
Dissociated Paneth cells loaded with secretory granules (SGs) in the small intestine (electron microscope: x5,000).

Electron microscopy revealed that the microvilli of ciliated enterocytes undergo a vesicular transformation. Intercellular contacts are disrupted, and enterocytes undergo dissociation (Figure 7). Edema, stasis, and erythrocyte sludges are observed in their respective layer.

The spleen is plethoric, with sinuses overfilled with erythrocytes. The red pulp contains hemosiderin granules and bilirubin crystals.

The general histological structure of the pancreas is preserved, though the changes have a mosaic pattern. In some areas, the epithelium of the excretory region is atrophied, and the interstitium is swollen, causing the lobules to separate.

Electron microscopy showed a decreased number of zymogenic granules in the apical parts of the secretory cells. The cisterns of the granular endoplasmic reticulum are dilated and swollen, with shortened cristae and increased transparency of the matrix.

Changes observed 24 hours after restoration of blood circulation

**Figure 8:**
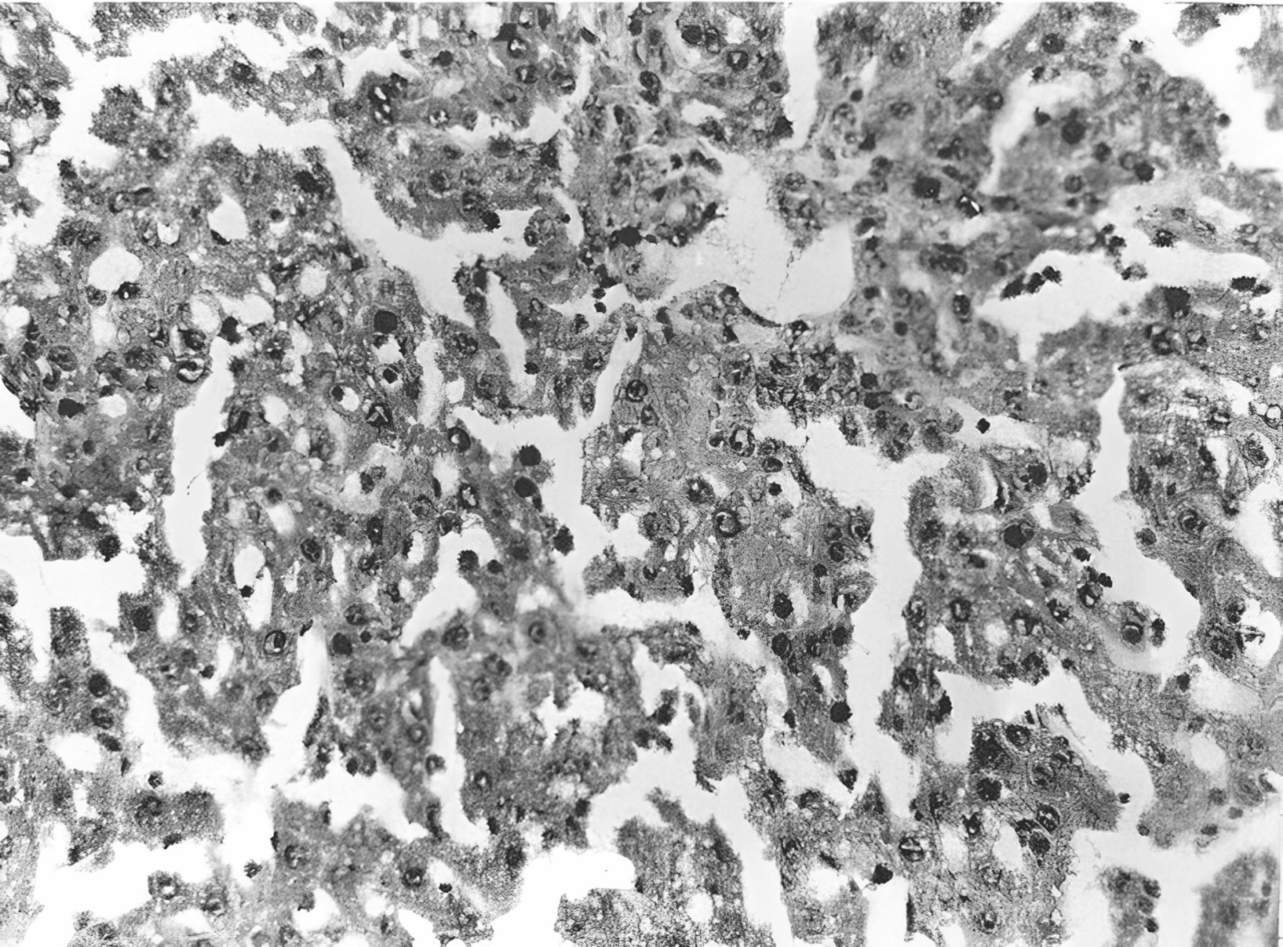
Dilation of the Disse spaces (light microscope, H&E stains: x240).

In H&E-stained preparations, the micromorphological structure of the liver is preserved. Disse spaces are dilated (Figure 8), and hepatocytes exhibit small- and large-droplet lipid dystrophy. Necrotic foci are rarely found. An increased number of apoptotic hepatocytes and proliferation of newly formed bile ductules (neoductules) are observed.

**Figure 9:**
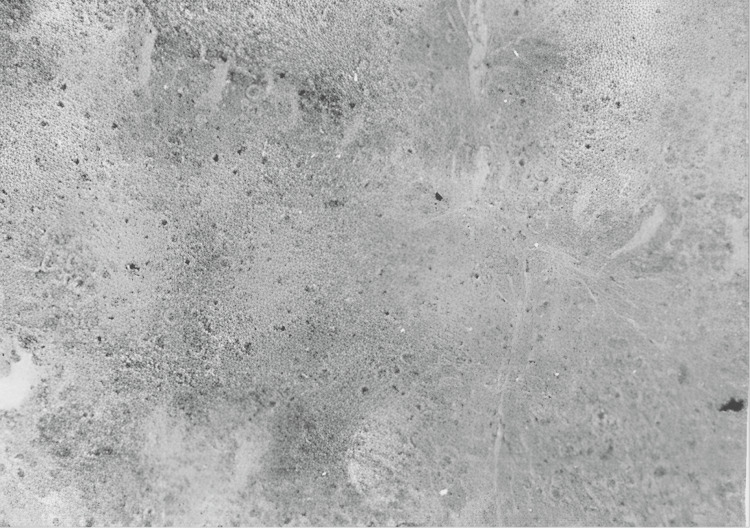
Shabadash's PAS reaction for glycogen modification is negative, with glycogen visible as a trace in some hepatocytes (light microscope: x120).

Mitotic figures are frequently observed. Glycogen is nearly absent in hepatocytes, except for a few in which only trace amounts are detectable (Figure 9).

**Figure 10:**
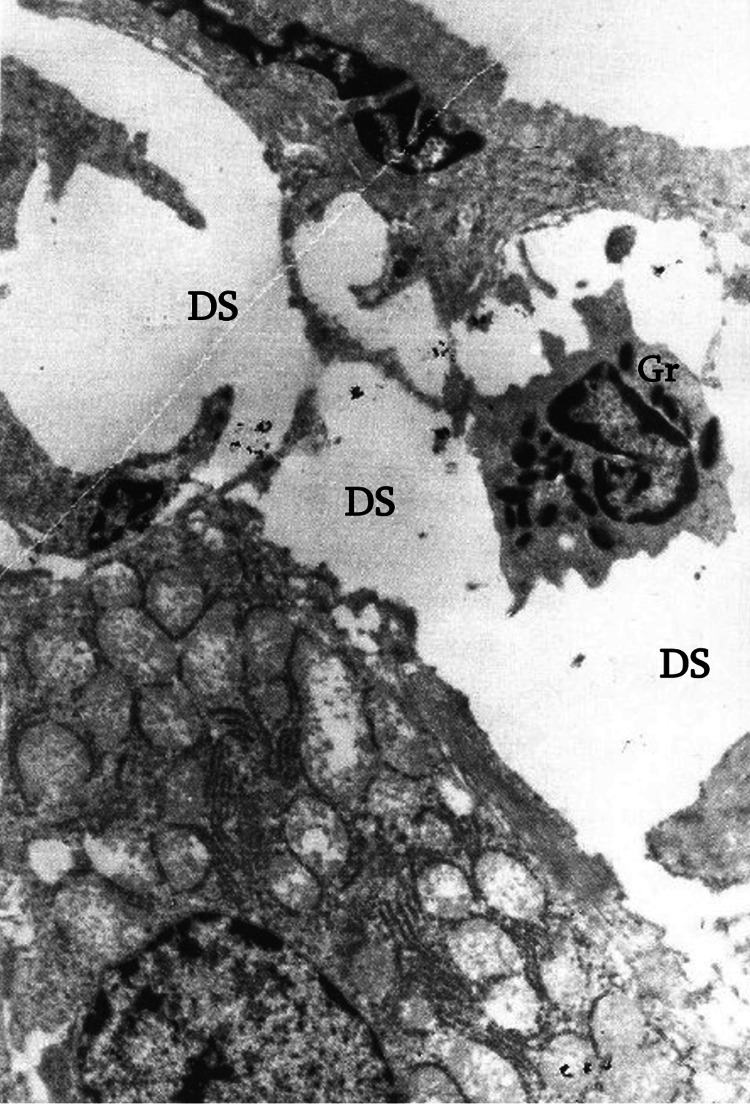
Dilation of the space of Disse (DS) and the presence of a granulocyte (Gr) within the space of Disse (electron microscope: x5,000).

Electron microscopy showed dilated (perisinusoidal) Disse spaces in the liver (Figure 10). Most hepatocytes exhibit severe ultrastructural changes, including marked mitochondrial vacuolization with cristae destruction, as well as vacuolization and degranulation of endoplasmic reticulum cisternae. Glycogen granules are absent.

**Figure 11:**
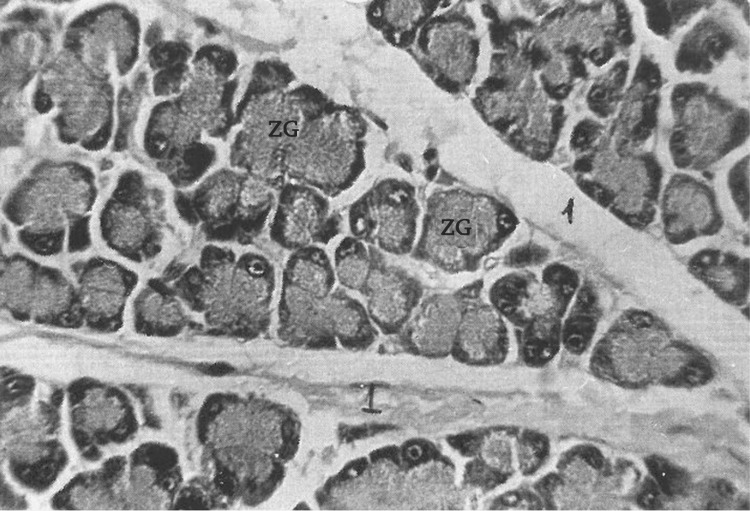
Acini of the exocrine pancreas are visible, with a structure nearly within the normal range, except for uneven loading of some pancreatocytes with zymogen granules (ZG) and the interstitium is slightly swollen (light microscope, H&E stains: x480).

In the pancreas, the general histological structure is preserved, and the changes display a mosaic pattern. Specifically, a decrease in secretory granules is observed in exocrine epitheliocytes, and in some cells with pyroninophilic granules, these granules are completely absent (Figure 11).

**Figure 12:**
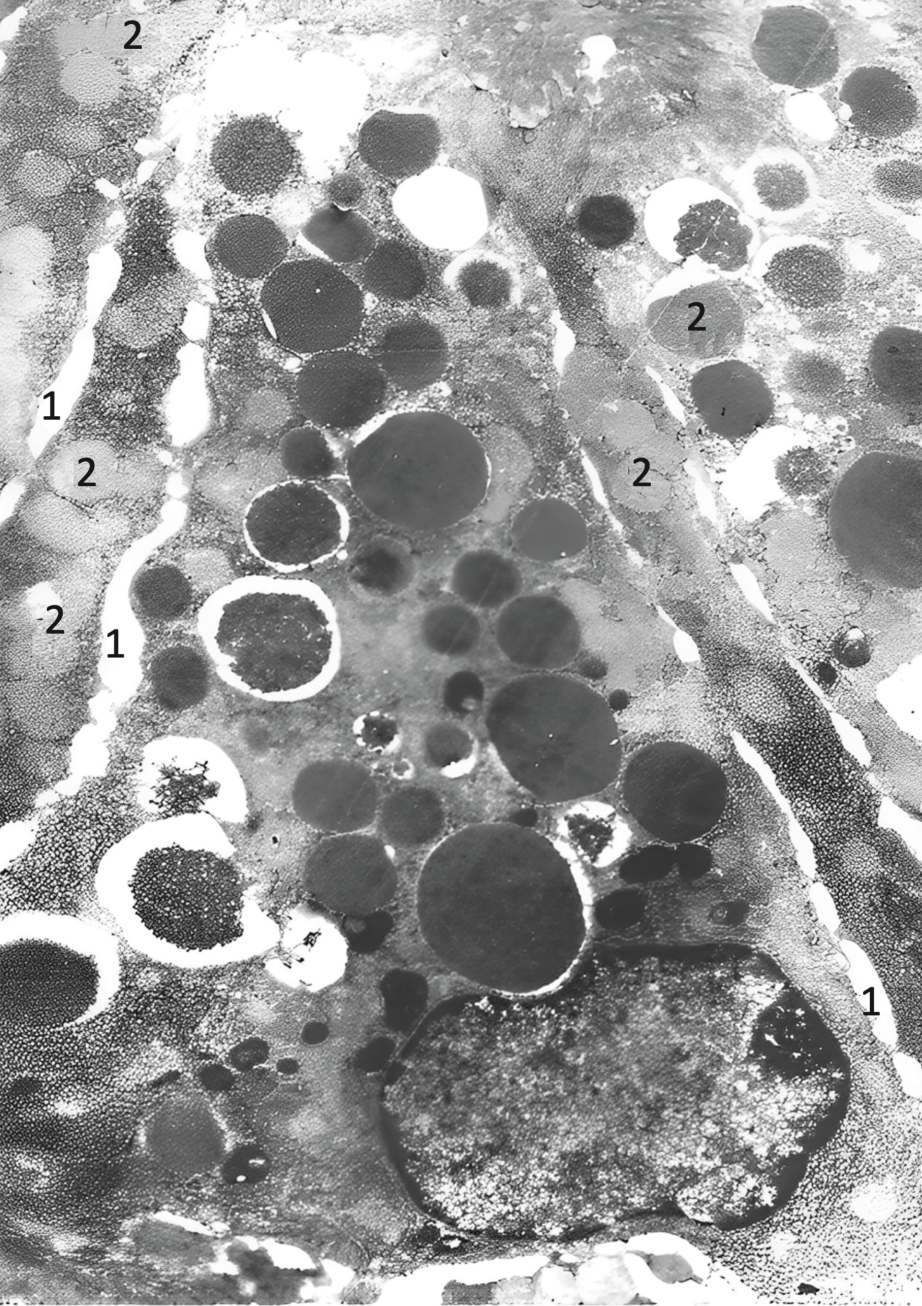
Dissociation of exocrine pancreatocytes (1) and apoptotic necrosis of pancreatocytes (2), whose cytoplasm is wrinkled and contains transparent zymogenic granules (2) (electron microscope: x10,000).

Electron microscopy revealed vacuolization of the endoplasmic reticulum cisternae in secretory cells, with partial degranulation in some areas. In the apical regions, the number of zymogen granules was reduced (Figure 12). Mitochondria appeared swollen, with flattened cristae. 

The small intestinal villi are swollen, with some stripped due to epithelial shedding. Electron microscopy revealed that the apical parts of the columnar epithelium are distended as a result of microvilli destruction and their transformation into vesicular structures, leading to enterocyte dissociation.

The spleen is plethoric. The sinuses are overfilled with erythrocytes. Moderate hyperplasia of the white pulp is observed. In the red pulp, foci of myelopoiesis and a considerable number of megakaryocytes are present.

Changes observed 48 hours after restoration of blood circulation

**Figure 13:**
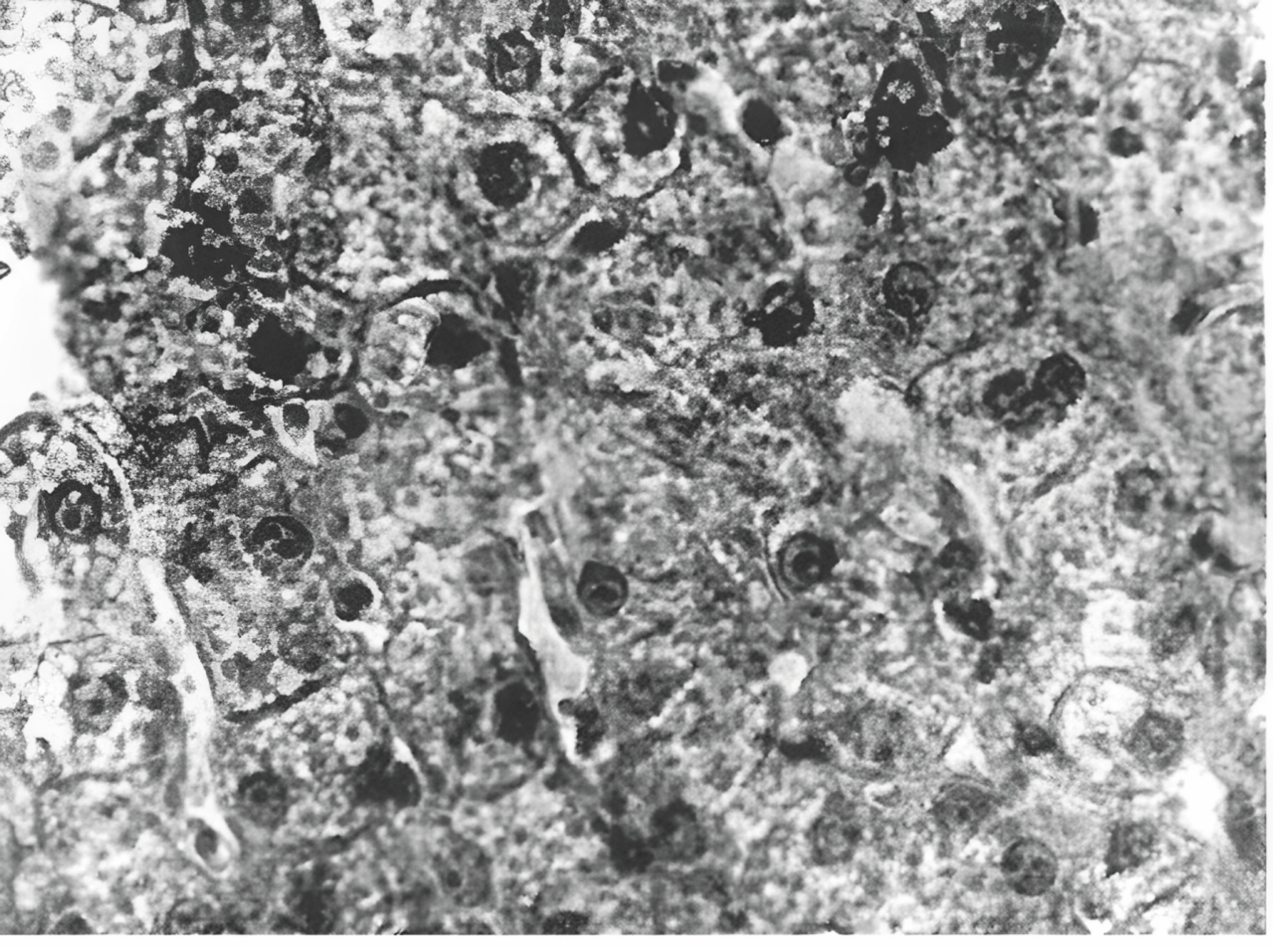
Relatively normal-shaped and swollen vacuolated hepatocytes visible in the liver (light microscope, H&E stains: x480).

**Figure 14:**
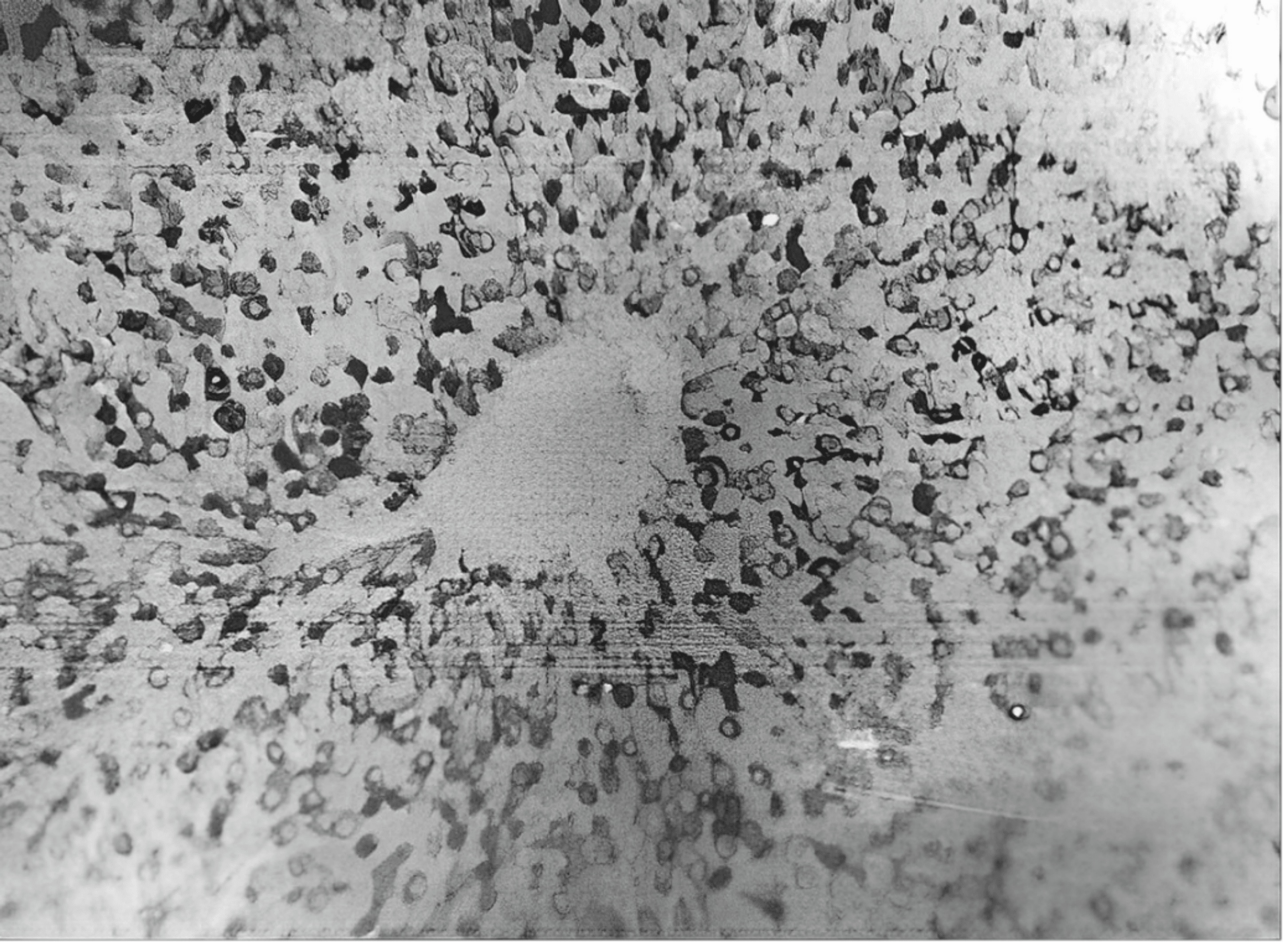
Shabadash's PAS reaction for glycogen (light microscope: x480) revealing glycogen-enriched hepatocytes are observed alongside glycogen-depleted hepatocytes.

In H&E-stained preparations, signs of hepatic damage are still present. Notably, some hepatocytes have regained a normal structure (Figure 13). In addition, a reduced amount of glycogens is observed in some hepatocytes (Figure 14). Mitotic figures are more frequently encountered compared to the 24-hour period.

**Figure 15:**
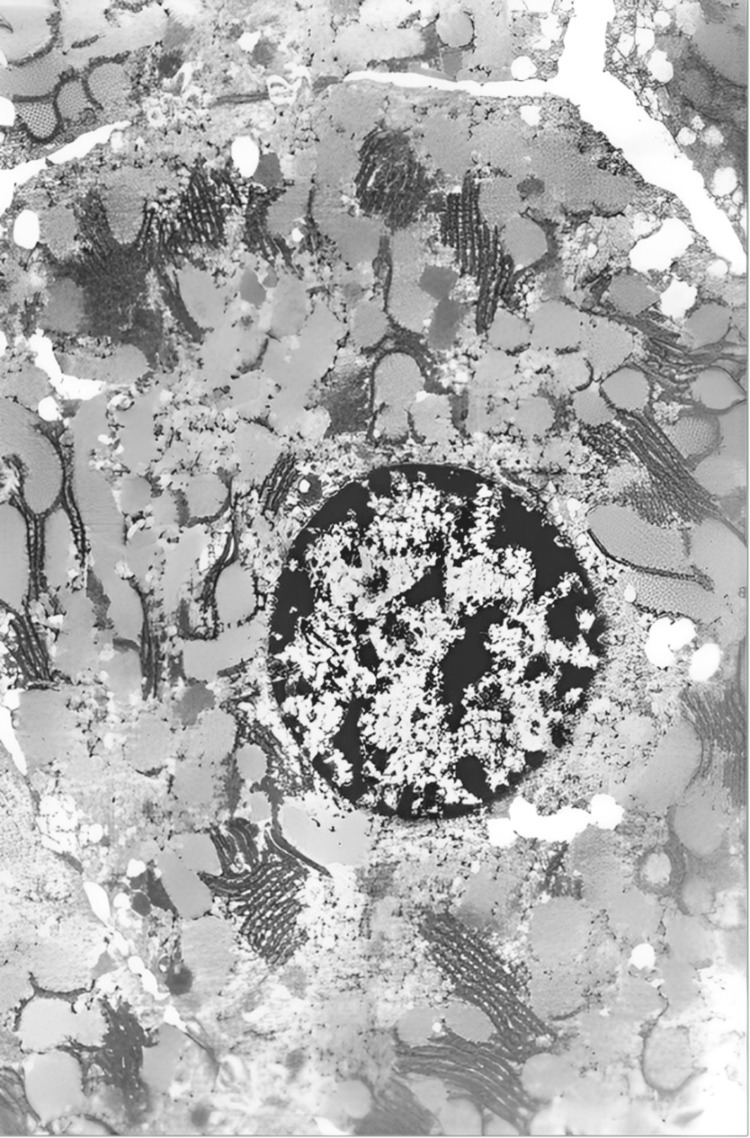
A hepatocyte, separated from the neighboring hepatocytes by a fissure-like space (dissociation), showing visible desmosomes in the area of the bile capillaries. The nuclear membrane is poorly defined, with heterochromatinization and marginalization of the chromatin. The cisternae of the endoplasmic reticulum contain protein material, while the smooth endoplasmic reticulum and glycogen granules are absent (electron microscope: x5,000).

Electron microscopy revealed distinct biliary and vascular regions in hepatocytes, with clearly visible microvilli. The cytoplasm contains a well-developed granular endoplasmic reticulum, whose cisternae are filled with protein material. The mitochondria are swollen, and the cisternae of the endoplasmic reticulum are vacuolated. Nuclear chromatin exhibits marginalization; nuclei are not clearly visible, and dissociation has begun. Cell connections are preserved only at the desmosomes in the area of the bile capillaries (Figure 15).

The general histological structure of the pancreas is preserved. The zymogenic regions of the pancreatic cells are densely packed with oxyphilic zymogen granules.

Electron microscopy revealed an abundance of secretory granules in the apical parts of the acinar cells. Small mitochondria are also visible, and the basal regions contain a well-developed granular endoplasmic reticulum.

In the small intestine, stripped villi are relatively rare; however, microfocal erosions are observed in some areas. Lymphoid follicles exhibit hyperplasia.

Electron microscopy showed that the absorptive enterocytes are covered with a luminal surface lined by a thin glycocalyx. Numerous pinocytotic vesicles are evident in the cytoplasm.

In the spleen, hemosiderin granules are visible in the red pulp. The lymphoid follicles display moderate hyperplasia.

## Discussion

After a temporary (10-minute) interruption of hepatic blood flow in the context of a six-day cholestasis, significant morphological changes developed in the liver and internal organs, particularly the small intestines, pancreas, and spleen. The changes varied depending on the time intervals (15 minutes, 24 hours, 48 hours) following the restoration of blood flow.

The extent of organ damage in cholestasis is characterized by its intensity, timing of development, duration, and underlying pathogenesis. Under cholestatic conditions, organ damage occurs much earlier than in the absence of cholestasis.

Therefore, immediately after the ligature is removed, severe changes are observed in the liver, particularly the areas of coagulative necrosis in its parenchyma. Such necrosis cannot occur under ischemic conditions alone; cholestasis, along with hepatocyte necrosis due to the pressure of stagnant bile in the intrahepatic biliary tract, may be contributing factors.

It is also an interesting fact that practically no traces of cholestasis are observed in the liver. This suggests that stagnant bile, having compromised the integrity of the biliary tract, escapes through the intrahepatic bile ducts and flows between the biliary capillaries and sinusoids, entering the bloodstream instead of returning to the liver. Notably, the proliferation of small bile ducts (“neoductulogenesis”) is observed as early as 15 minutes and 24 hours after the restoration of blood circulation, but reverses in 48 hours [3-9].

Severe changes also occur in the intestines, pancreas, and spleen, appearing more severe and last longer than in the absence of cholestasis. This suggests that the changes observed in the liver and other internal organs under cholestatic conditions have an ambiguous role in contributing to the overall irreversibility of the organism’s condition.

We hold a distinct view on neoductulogenesis, particularly regarding the role of biliary hypertension as a primary cause. We propose that neoduct formation does not result from the proliferation and branching of interlobular bile ducts, but rather from the transformation of bile capillaries located between intralobular hepatocytes into bile ducts. Specifically, when biliary hypertension develops within the interlobular bile ducts, the resulting biliary congestion extends to the intralobular bile capillaries. This appears to signal hepatocytes to reduce bile production and begin transforming into bile duct epithelium, cells capable of synthesizing their own basement membrane, proliferating, and forming new bile ducts, or "neoductules." Our hypothesis on the origin of neoductules is supported by other researchers [10,11], who have identified these unique liver cells as exhibiting phenotypic characteristics intermediate between hepatocytes and intrahepatic biliary epithelium. It has been suggested that parenchymal hepatocytes can transform into bile duct epithelium and vice versa. A similar process is believed to occur in the pancreas [12].

Although the liver is initially the most significantly affected organ, and the primary pathological changes occur there, it is also capable of relatively rapid structural and functional recovery due to its high regenerative capacity, compared to other organs [13-16].

Study limitations

However, several limitations should be acknowledged in this study.

Duration of Observation

The current observation intervals following blood flow restoration (15 minutes, 24 hours, 48 hours) may not fully capture the long-term consequences of liver disconnection and reperfusion. Future studies would benefit from extended observation periods to more accurately assess the full extent of liver and systemic recovery or deterioration.

Controlled Variables

Other variables, such as the animal's age, underlying health conditions, and environmental conditions, were not controlled, potentially influencing the outcomes. Implementing a more controlled experimental setting could help minimize these confounding factors.

Histological Methods

While the histological methods employed in this study offer valuable insights, they are limited in capturing the full spectrum of molecular and cellular changes. Incorporating advanced molecular and genomic techniques could yield a more comprehensive understanding of the underlying pathophysiological processes.

## Conclusions

Under cholestatic conditions, the damaging changes in the liver and other organs following temporary interruption of hepatic blood circulation and its restoration at different time intervals last longer and are more severe than in the absence of cholestasis. Fifteen minutes after ligature removal, new bile duct formation or “neoductulogenesis” is observed, providing an additional reservoir for congested bile. However, 24 and 48 hours after ligature removal, a sharp decrease in the number of neoductules occurs. This reduction is driven by the flow of congested bile into the bloodstream through new pathways, drainage of intrahepatic bile ducts, and reduced intrahepatic bile pressure, factors that likely stimulate metaplasia.

The primary cause of the morphogenic changes and disruption of homeostasis following temporary interruption of hepatic blood circulation and reperfusion is not ischemic liver damage, as previously believed, but a circulatory disorder resembling hypovolemic shock. This condition arises due to congestion in the portal vein system and is characterized by hypoxic damage to peripheral organs and the development of a “Locked-in syndrome“ after reperfusion. To prevent bleeding during large-scale liver surgeries, we recommend the effective use of the tourniquet method on the hepatoduodenal ligament, both preoperatively and during surgery. Additionally, in the postoperative period, attention should be given not only to the morphofunctional state of other organs but also to the implementation of appropriate preventive and curative measures, ensuring full preparedness to address any organ dysfunction.
